# Rich observations of local and regional infrasound phases made by the AlpArray seismic network after refinery explosion

**DOI:** 10.1038/s41598-019-49494-2

**Published:** 2019-09-10

**Authors:** Florian Fuchs, Felix M. Schneider, Petr Kolínský, Stefano Serafin, Götz Bokelmann

**Affiliations:** 1 University of Vienna, Department of Meteorology and Geophysics, Vienna, 1090 Austria; 2 Helmholtz Centre Potsdam, GFZ German Research Centre for Geosciences, Potsdam, 14473 Germany; 3 University of Innsbruck, Department of Atmospheric and Cryospheric Sciences, Innsbruck, 6020 Austria

**Keywords:** Atmospheric dynamics, Seismology

## Abstract

On September 1st, 2018 a devastating explosion occurred on the facility of an oil refinery near Ingolstadt, Germany. We analyzed data of 400 permanent and temporary seismic stations and find strong seismo-acoustic signals on more than 80 seismic stations. The infrasound signal is detectable on seismic stations within 10–350 km from the source, with 40 km spatial resolution. We confirm the explosion site both by the seismic and seismo-acoustic arrivals. Apart from seismic P- and S-waves, we identified three separate acoustic phases with celerities of 332, 292, and 250 m/s, respectively, each of which has a particular spatial pattern of positive detections at the ground. Seismo-acoustic amplitudes are strongly affected by the type of seismic installation but still allow insight into regional infrasound attenuation. Our observations likely represent tropospheric, stratospheric, and thermospheric phases. We performed 3D acoustic ray tracing to validate our findings. Tropospheric and thermospheric arrivals are to some extent reproduced by the atmospheric model. However, ray tracing does not predict the observed acoustic stratospheric ducts. Our findings suggest that small-scale variations had considerable impact on the propagation of infrasound generated by the explosion.

## Introduction

Dense seismic networks such as the transportable USArray or the European AlpArray^1^ enable scientist to study the propagation of seismic waves in unprecedented spatial detail. While they are designed to record and analyze seismic wavefields with great precision^2–4^, recent studies highlight the ability of seismic networks to also detect and track acoustic signals over distances up to hundreds of kilometers^5,6^. As dedicated infrasound installations are rare anywhere on earth, this renders seismic arrays and dense regional seismic networks particularly useful to study the infrasound signals generated by strong explosive events, both of natural^7–10^ and man-made origin^6,11–13^. Data from an accidental rocket explosion recorded by the transportable USArray highlighted the potential wealth of information that could be gained from spatially dense infrasound records^14^.

Here we report on a devastating explosion that occurred on September 1st, 2018 near Ingolstadt, Germany. The explosion has created both seismic and acoustic waves that were detected by seismic stations up to 400 km distance. The dense spatial station coverage of the AlpArray seismic network allows to study the propagation of both the seismic and the acoustic wave field in great detail. Here, we focus in particular on the seismo-acoustic observations, that reveal a unique number of various seismo-acoustic phases detected on regional scale and potentially allow for improved insight into local and regional infrasound propagation and into seismo-acoustic coupling.

## Observations

### Seismo-acoustic data

**Figure 1:**
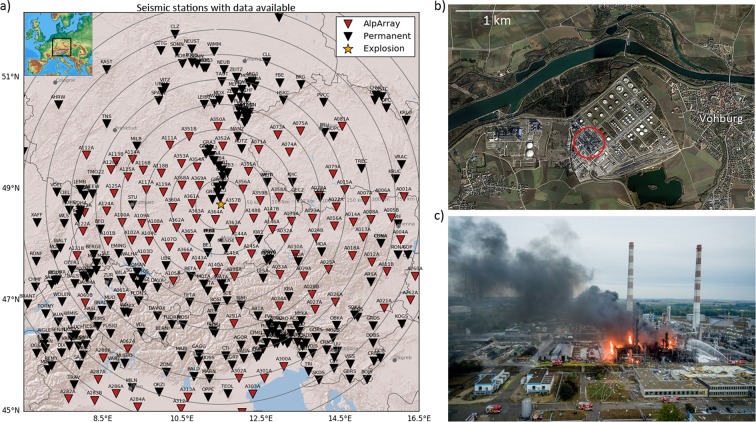
(**a**) Map of all seismic stations used in this study. Red triangles mark temporary AlpArray installations, black triangles mark permanent seismic stations. The inset highlights the location within Europe. The map image was created using the Matplotlib Basemap toolkit version 1.0.7: https://matplotlib.org/basemap/ (last accessed August 2019). (**b**) Aerial view of the refinery site (Map data: Google, GeoBasis-DE/BKG). The part where the explosion occurred is highlighted. (**c**) Photograph of the damage after the explosion (https://paf-kick.de/443-fci-040918, last accessed August 2019, reproduced with permission from M. Matthes, THW Pfaffenhofen).

According to news reports the explosion occurred on September 1st, 2018 between 03:00 and 03:15 UTC at the site of an oil refinery operated by BayernOil near the village of Vohburg in Bavaria, Germany (Coordinates: 48.766, 11.595). Local newspaper report that the explosion was audible in several kilometers distance and caused severe damage to buildings in neighboring villages^15^. According to reports, hot fuel escaped through a crack from a highly pressurized (25 bar) container and eventually got ignited. Structural damage near the explosion indicates that the explosion occurred above ground. The explosion site is located 12 km east of Ingolstadt and 10 km south of the Gräfenberg broadband array^16^ and is well-covered by temporary broadband stations of the AlpArray seismic network^1^ with station spacings of approximately 40 km (see Fig. 1). The AlpArray stations provide excellent spatial coverage for this event, in particular as Bavaria in southern Germany is only sparsely instrumented with permanent sensors. Additionally, most AlpArray stations provide data in real time, so timely analysis of the explosion data was possible.

We acquired data from a total of 400 seismic broadband stations within 400 km radius of the explosion site (Fig. 1). Waveform data and associated metadata were downloaded from the European Integrated Data Archive (EIDA). We focused exclusively on vertical component data, with 100–200 Hz sampling rate. All data were corrected for the instrument response to obtain ground velocity records. We manually inspected the data for impulsive signals that are potentially related to the explosion, and travel at seismic velocities (2–8 km/s) as well as acoustic velocities (≈0.33 km/s).

**Figure 2:**
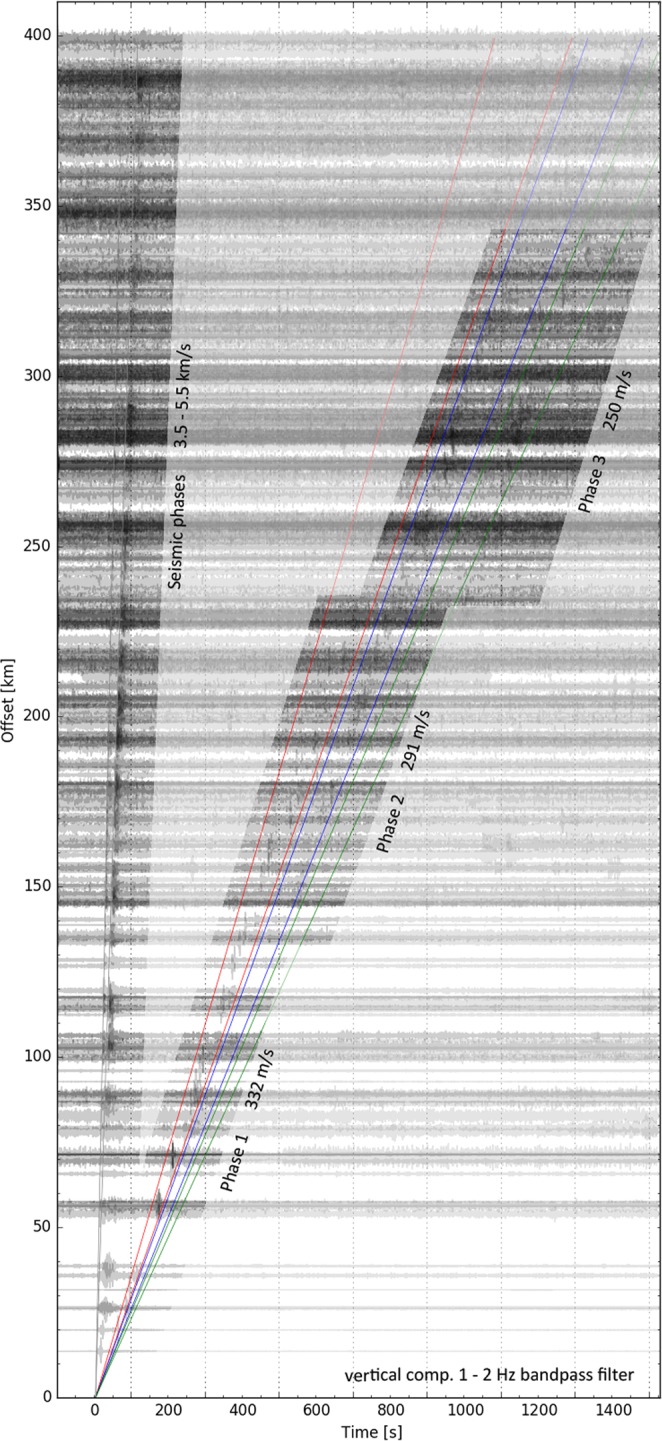
Record section showing the vertical component ground velocity for all 400 seismic stations, bandpass-filtered between 1–2 Hz (that provides the best visual signal-to-noise ratio). All traces are displayed and no selection was made. Each trace/station is normalized to its individual maximum. The seismic and the three separate acoustic phases are highlighted. Time 0 denotes the origin time of the explosion (03:11:45 UTC). The search windows for phases 1, 2 and 3 are marked by red, blue and green lines, respectively. The corresponding velocities are: Phase 1 310–340 m/s, Phase 2 270–300 m/s, Phase 3 240–260 m/s (see also Table 1).

Figure 2 shows a record section of all available data up to 400 km from the explosion. We identified both seismic P and S phases up to a distance of 400 km (the maximum extent of our study area). They are traveling with an apparent velocity of approximately 5.5–5.9 km/s and 3.5 km/s, respectively. Additionally, three separated phase arrivals are visible at times between 50 and 1300 seconds after the explosion and up to 350 km distance. They appear most prominently in the 1–2 Hz frequency range (see Fig. S1, electronic supplement, for a comparison of different frequency bands). These phases travel with velocities of 310–340 m/s (1st phase; visible at 0–200 km distance), 270–300 m/s (2nd phase; visible at 150–325 km distance) and 240–260 m/s (3rd phase; visible at 250–350 km distance) (see also Table 1). There is considerable offset between neighboring phases (≈120 s between phases 1 and 2; ≈160–180 s between phases 2 and 3), and within certain distance ranges (e.g. 150–175 km and 250–350 km) more than one phase is visible. These velocities strongly suggest they represent seismo-acoustic signals that were generated by the refinery explosion.

**Table 1:** Summary of seismo-acoustic signal detections for the three observed acoustic phases.

	Dist. range/km	Cel. range/m s^−1^	Mean cel. ± Std./m s^−1^	Stations	AlpArray
Phase 1	0–200	310–340	332.2 ± 8.7	40	23
Phase 2	150–325	270–300	291.7 ± 5.3	25	3
Phase 3	250–350	240–260	250.1 ± 5.7	19	9
			Total	84	35

Distance range marks distances to the source in which the respective phase was observed. Celerity (cel.) range states the respective celerities that were used to search for acoustic arrivals. Mean and standard deviation of celerities are calculated for all detected arrivals with signal-to-noise ratio higher than three. The columns “Stations” and “AlpArray” denote on how many stations the signal-to-noise ratio was measured higher than three (total number of stations and number of temporary AlpArray stations therein, respectively).

The seismo-acoustic explosion signal is detected by a large number (>80) of seismic stations, complicating any manual analysis of single-station data. Thus, we defined objective criteria to assess if the signal is positively detected at a given station. Based on the observations described above we defined three separate arrival time windows, calculated using the observed effective velocities: Phase 1, 310–340 m/s; Phase 2, 270–300 m/s; Phase 3, 240–260 m/s. For each time window we determine the maximum absolute value of the vertical peak ground velocity (PGV) in the 1–2 Hz frequency band, and the corresponding celerity (=total travel time from source to receiver divided by horizontal distance). Background noise is calculated as three times the standard deviation (3*σ*) of seismic amplitudes (in the 1–2 Hz band) within a 60 s window that ends 20 s before the explosion. If the measured vertical PGV exceeds the background noise by a factor of three we label the respective station as a positive detection. This way we identify the seismo-acoustic signal on in total 84 different seismic stations within 350 km from the explosion site. 35 of these are temporary AlpArray installations. None of the stations shows high SnR signals of more than one individual phase. Note that potentially more stations may show weaker but still manually recognizable signals of the explosion and secondary phases. These are however not considered in this study. Table 1 summarizes the number of detections for the three different phases, as well as the calculated celerities. Figure S2 displays the distribution of all individual arrival times and calculated celerities as read from the PGV in each time window.

### Spatial distribution of detections

**Figure 3:**
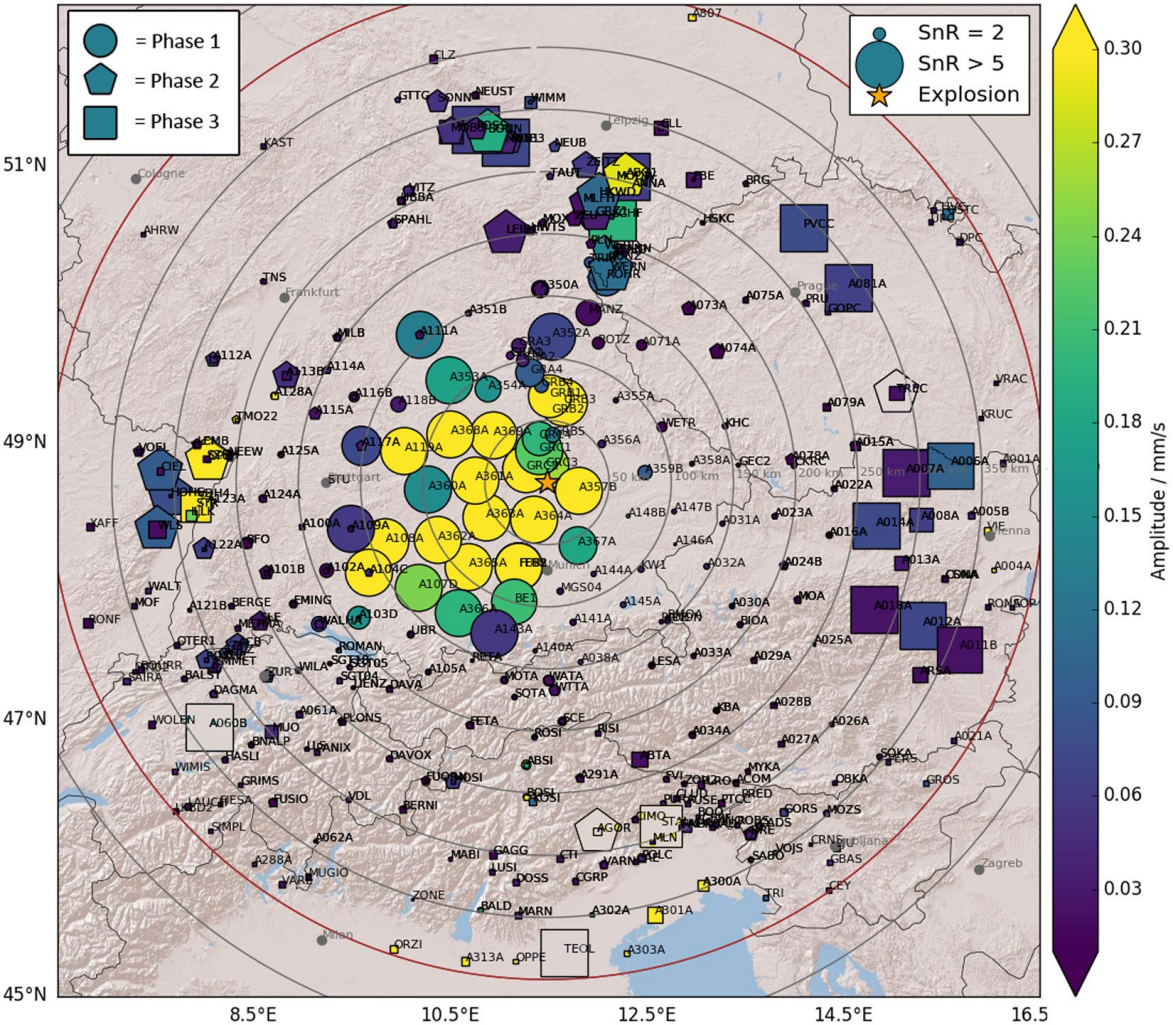
Maximum amplitudes of the seismo-acoustic phases on all stations. The three separate acoustic phases are denoted by different symbols (circle = phase 1, pentagon = phase 2, square = phase 3). All symbols are colored by amplitude (PGV) and scaled by the signal-to-noise ratio (SnR). This way, stations that recorded the seismo-acoustic signal with high SnR are visually highlighted and others are suppressed. Note that the color scale saturates at a PGV of 0.3 mm/s and that the size of the symbols is truncated at a SnR of 5. Some of the isolated high-SnR markers are likely false positive detections due to strong noise in the respective time window and are not colored. Please see Fig. S3 for separate maps of each acoustic phase. The map image was created using the Matplotlib Basemap toolkit version 1.0.7: https://matplotlib.org/basemap/ (last accessed August 2019).

The positive detections of the seismo-acoustic explosion signal show pronounced spatial patterns that are significantly different among the three separate phases. Figures 3 and S3 show the spatial distributions of amplitudes for all three phases. Phase 1 is predominantly observed in southerly to northwesterly directions (135–315° azimuth from the source) at distances up to 200 km (see Figs 3 and S3a). A branch of weak signals also potentially stretches out northwards past stations of the Gräfenberg array up to distances of almost 250 km. Seismo-acoustic phase 2 is recorded at distances between 150–325 km, and in narrow corridors towards North (330–45°) and West (240–300°) and is absent elsewhere (Figs 3 and S3b). Phase 3 is recorded in a broad azimuth range (350–115°) from North to South-East of the explosion site at distances of 250–350 km (see Figs 3 and S3c). Note, that within the distance ranges of phases 2 and 3 (beyond 150 km distance) the spatial station coverage is greatly reduced into north-western directions (beyond the extent of the AlpArray seismic network) and north-eastern directions (AlpArray stations existing, but no real-time data available).

### Location: seismic vs. acoustic

In order to independently locate and time the source of the detected signals we identified 9 stations that show the earliest arrivals of both the seismic and the seismo-acoustic phase. For these stations we manually picked the first onset of the seismic P-arrival and the first onset of the seismo-acoustic arrival (see Figs S4 and S5, supplement and Table 2). Both sets of onset readings were independently used for a simple grid search routine that determines the epicenter and origin time of the explosion by minimizing the root-mean-squared residuals between observed and theoretical arrival times of all stations simultaneously. Grid points are spaced by 1 km and time is searched in steps of 0.1 s for seismic waves and 1 s for acoustic waves. We obtained an estimate of the propagation velocities through linear regression along the picked first onset, assuming that the velocity is constant and isotropic. This resulted in a propagation velocity of 5.9 km/s for the seismic P-waves and 335 m/s for the acoustic waves (see Fig. S2).

**Table 2:** Values for determination of the explosion origin time based on theoretical acoustic travel times.

Station	Dist./km	Azim./Deg.	Pick/UTC	Bounce dist./m	Cel./m/s	Travel time/s	Origin time/UTC
GRC3	14	357	03:12:27.30	–	–	–	–
GRC2	20	305	03:12:43.88	149	338	58.6	03:11:45.25
GRC1	26	348	03:13:04.82	–	–	–	–
A357B	30	104	03:13:06.34	–	–	–	–
A364A	31	200	03:13:15.40	305	339	94.5	03:11:40.94
GRB5	38	9	03:13:45.59	–	–	–	–
A363A	56	241	03:14:26.02	654	340	167.8	03:11:38.17
A369A	57	313	03:14:35.11	–	–	–	–
A361A	57	273	03:14:32.44	463	339	169.8	03:11:42.60
FFB2	71	200	03:15:11.14	848	340	212.2	03:11:38.88
A368A	86	298	03:16:00.38	870	338	254.7	03:11:45.63
						Mean ± Std. dev.	03:11:41.9 ± 2.8 s

Distance (dist.) denotes the distance from the explosion site. Azimuth (Azim.) indicates the azimuth from the explosion to the respective station. Bounce distance denotes the distance from the station to the closest calculated surface bounce point. el. denotes the modeled celerity. Note that several stations on the list correspond to the stations used for the location grid search, but have no matching bounce point available from ray tracing.

**Figure 4:**
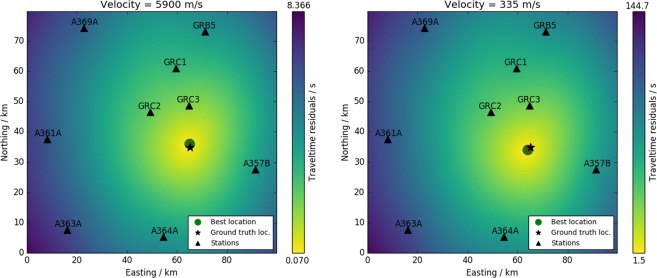
Comparison of location results using the seismic P phase (**a**) and the acoustic phase (**b**). Black triangles mark the stations used. Color denotes the smoothed travel time residuals for each point of the underlying grid (1 km grid size). This is the snapshot for the best-fitting origin time. The green dot highlights the best-fitting epicenter location, compared to the ground truth epicenter (star). The red ellipses denote the area in which the travel time residuals are less than 10% higher than at the minimum. Seismic and acoustic results are similar in quality.

Figure 4 compares the location results based on seismic and acoustic phases. Using the seismic phases we locate the source 1 km (i.e. one grid point) north of the true origin. Using the acoustic phases we locate the source 1.5 km (i.e. one grid point along diagonal) south-west of the true origin. This clearly confirms the refinery site as the epicenter of both the seismic and the seismo-acoustic signal. Relative to the mean total travel time, the remaining travel time residuals for the best epicenter solution are similar for the two approaches (1.5 s/108 s ≈ 1.4% acoustic; 0.07 s/7.6 s ≈ 0.9% seismic). The extension of the 10%-error ellipse is small (<1 km) both for the seismic and acoustic result (see Fig. 4). Based on the acoustic picks, we determine the origin time of the explosion to 03:11:44 UTC, and based on the seismic picks the the best-fitting origin time is 03:11:46 UTC. Thus we define the origin time to the mean value of 03:11:45 UTC ± 1 s.

The explosion is listed in the event bulletin of the International Seismological Centre (ISC, event ID 612689977^17^) with an origin time of 03:11:45 UTC, in agreement with our findings. The hypocenter is located at the surface and 2.5 km south of the refinery site. The local magnitude is M_*l*_ = 2.0 and the body wave magnitude is m_*b*_ = 1.9.

### First onset polarities

On the same stations as used for the localization we also analyzed the polarity of the first onset for both the P-wave and the seismo-acoustic wave. For this purpose data was not filtered between 1–2 Hz as for most of the other measurements, but with a wide passband of 0.1–25 Hz to avoid artificial disturbance of the waveforms (note that the lower corner frequency of 0.1 Hz is far below any frequencies contained in the signal. Without any filtering, the onsets are hardly visible on several stations due to strong noise. For consistency we applied the filter even to the low-noise stations). All P-wave onsets show a clear positive vertical first motion (upwards), regardless of the azimuth between station and source (see Fig. S4, supplement). In contrast, the onsets of the seismo-acoustic waves are generally less pronounced and do not show a regular pattern (see Fig. S5, supplemental), with some stations showing clear downward first motion and others suggesting upward first motion, with no correlation to the azimuth.

### Seismo-acoustic amplitudes and attenuation

**Figure 5:**
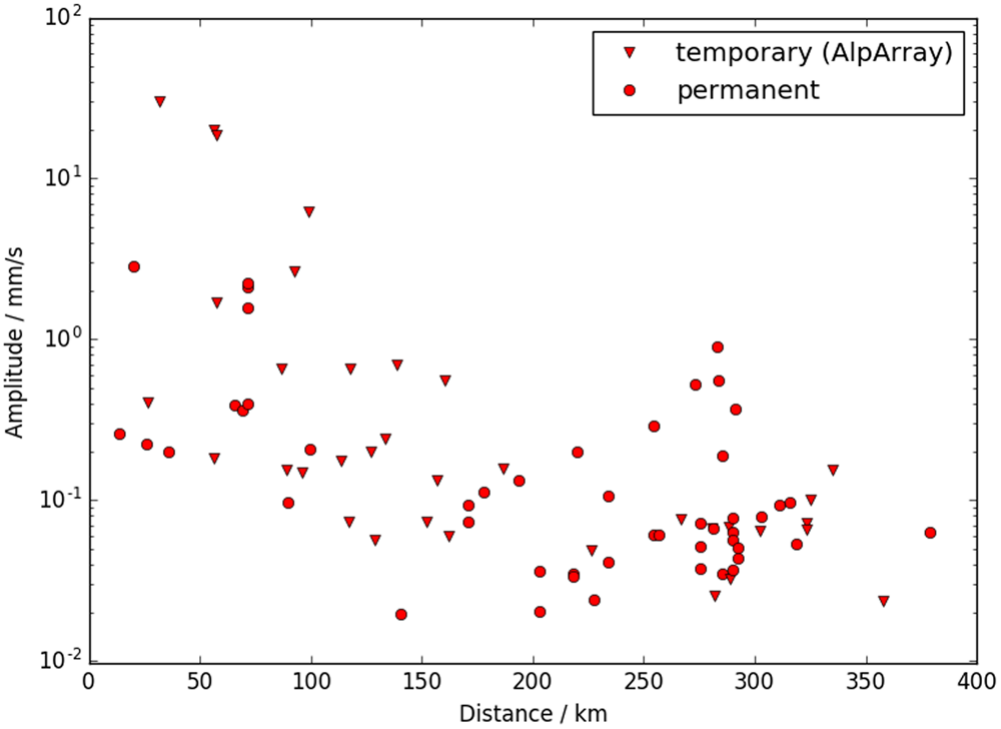
Maximum amplitude of the seismo-acoustic signal (all three phases) versus distance from the source. Temporary and permanent seismic stations are denoted by triangles and circles, respectively. Only measurements with a signal-to-noise ratio larger than three are plotted.

The dense spatial coverage around the explosion site allows some potential insight into the attenuation of the seismo-acoustic signal with distance. For this analysis we only considered stations with a signal-to-noise ratio of larger than three (in the 1–2 Hz frequency band). Figure 5 displays the maximum vertical amplitude of the seismo-acoustic signal with distance. Our data show large variations in seismo-acoustic amplitudes, but on average within 200 km from the source the observed seismo-acoustic amplitudes attenuate by approximately 1–2 orders of magnitude. Notably, we observe that seismo-acoustic amplitudes are generally higher on the temporary AlpArray installations compared to the permanent seismic stations. Within 25–50 km from the source, the PGV induced by the seismo-acoustic signal at AlpArray stations is (on average) more than 70 times larger than average PGV measured on permanent stations (e.g. the Gräfenberg array). Within 50–150 km from the source the seismo-acoustic mean PGV measured on temporary AlpArray stations is still 10 times higher than on permanent stations at the same distance from the source. Factors that impact the seismo-acoustic amplitudes are reviewed in the discussion section.

### Acoustic raypath modeling

The previous observations clearly indicate that we are observing a seismo-acoustic phenomenon, and that the refinery explosion created strong infrasound signals. Thus, similar to the approach in^6^ we modeled the propagation of infrasound rays from the source up to 400 km distance, in order to compare modeled surface bounce points with our observations. We used the GeoAc ray tracing suite^18,19^ to perform 3D ray tracing in a proper atmospheric model.

We extracted the required atmospheric parameters (wind speed, wind direction, temperature, etc.) from an hourly updated forecast model provided by the European Centre for Medium-Range Weather Forecasts (ECMWF). We retrieved the 3D ECMWF model for 2018/09/01 03:00 UTC on a grid ranging from 45° to 52° longitude and from 7° to 17° latitude with 0.125° resolution. For distances up to 400 km from the source thermospheric arrivals are expected, so we extended the ECMWF model to higher altitudes using the MSISE-00^20^ and HWM14^21^ empirical models.

To guarantee a smooth transition between ECMWF and HWM14/MSISE-00 models we mixed the models between 67 and 77 km altitude. Below 67 km altitude we used the pure ECMWF model and above 77 km altitude the pure model derived from MSISE00 and HWM14. A linear transition between the models is implemented in the 67 km–77 km range. The resulting merged input model for GeoAc (calculated using the barometric formula) measures height above sea level (with z = 0 being sea level). However, as infrasound only propagates in air above ground, we subtracted the local topography at each grid point from the height column such that for each grid point the height measures start at ground level (z = 0 being ground level instead of sea level).

**Figure 6:**
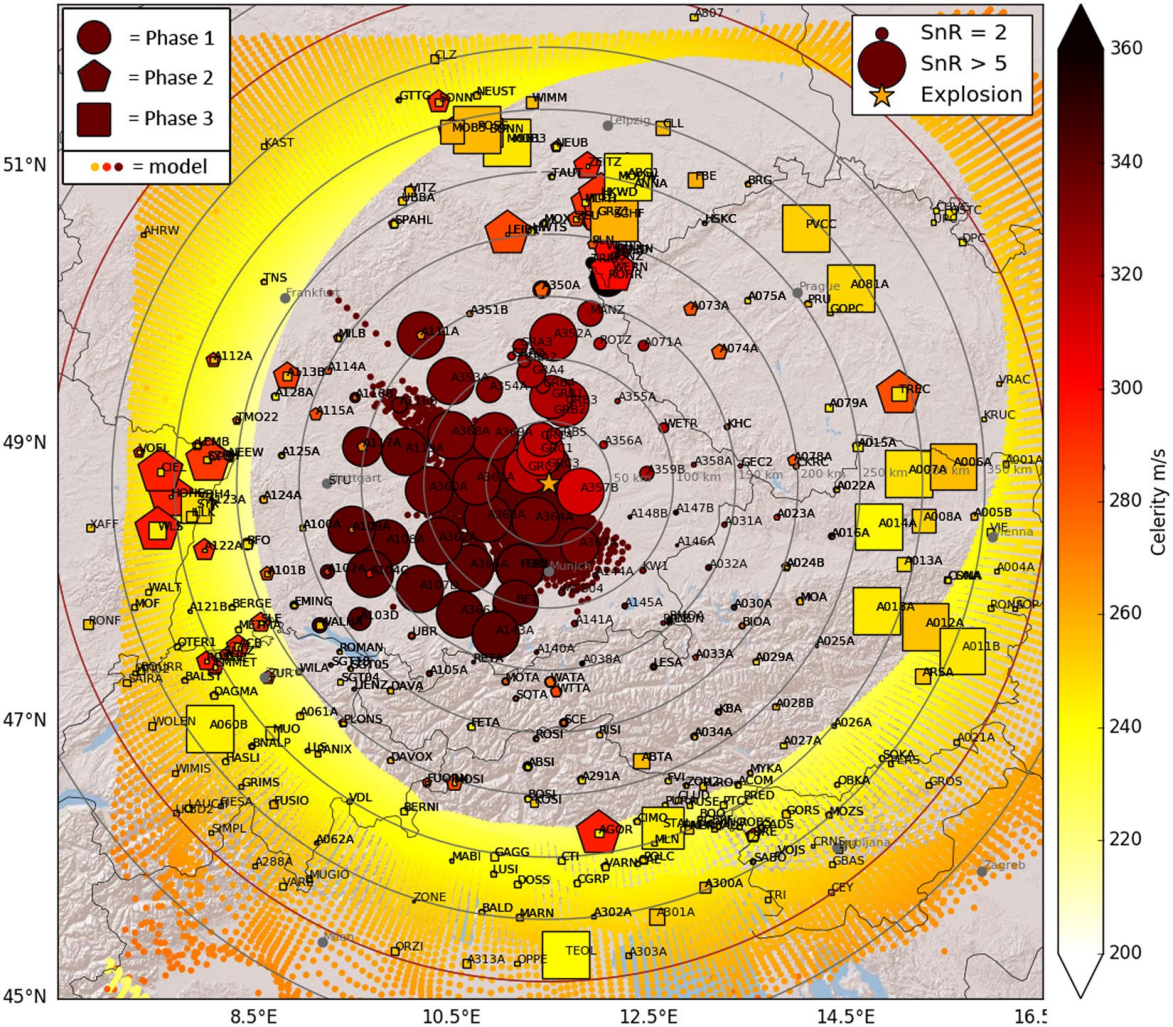
Measured versus modeled celerity. The color of the large symbols denotes the measured celerity of the respective arrival. Symbols are scaled in size by the signal-to-noise ratio (SnR) of the seismo-acoustic signal. Small colored dots indicate the calculated surface bounce points and their respective celerity as obtained from the acoustic ray tracing. The map image was created using the Matplotlib Basemap toolkit version 1.0.7: https://matplotlib.org/basemap/ (last accessed August 2019).

Figure 6 shows the surface bounce points resulting from the 3D acoustic ray tracing, colored by the expected celerity, and compares the modeling results to our measurements. The acoustic ray tracing predicts a high-celerity tropospheric duct resulting in several bounce points within 0–150 km distance from the source, in the 130–300° azimuth range. Notably, the model does not predict any stratospheric ducts and consequently no surface bounce points with medium celerities. Thermospheric reflections with low celerity are expected at all azimuths, yet at different distances depending on azimuth. All calculated thermospheric reflections show celerities increasing with distance. Please refer to the discussion section for a detailed comparison of measurements and modeling.

The ray tracing results predict precise travel times for several seismic stations that co-locate with calculated tropospheric bounce points (see Table 2). We deduct this theoretical travel time from the observed first onset pick to independently determine the origin time of the explosion. Measurements from six stations within 20 and 86 km distance result in a mean origin time of 03:11:41.9 UTC ± 2.8 s (see Table 2 for details), slightly earlier than estimated by the seismic and acoustic grid search, but still within the estimated uncertainties. Note, however, that we could not use the same set of stations as for the grid search, because the modeling did not reproduce any bounce points north of the explosion. All stations with modeled travel times available are exclusively south/west of the explosion, potentially also introducing spatial bias.

## Discussion

### Seismo-acoustic detections

The seismic and seismo-acoustic signals we have observed are clearly associated with the refinery explosion near Ingolstadt, Germany on September 1st, 2018. The origin times determined from seismic and acoustic grid search as well as the ray tracing agree within their respective uncertainties. Additionally, all observed acoustic phases show a stable and steady move-out from the source with distance, evidence by well-aligned acoustic arrival times and little scatter in measured celerities (see Fig. S2). Thus, we are confident that all signal-to-noise ratio based detections for phases 1 and 2 and also most of the detections for phase 3 are in fact due to the refinery explosion and not due to local noise sources. Additionally, we searched the publicly accessible seismic event bulletin of the Swiss Seismological Service (SED), the earthquake service for Bavaria (LMU), the monthly E-Mail event bulletin for Austria (ZAMG) and the ISC bulletin for seismic events that occurred close in time to the Ingolstadt explosion. Except the explosion itself (registered in the ISC event bulletin) there are no seismic events registered within 400 km from the explosion site and within hours after the explosion. Consequently we can rule out that e.g. the late arrivals of phase 3 are due to other seismic events. Closer inspection of the seismic record sections also shows no indications for potential secondary infrasound sources (e.g. from topography^22^).

Each of the observed seismo-acoustic phases displays a different celerity, indicating that they each follow an individual propagation path. The values of 332 m/s (phase 1), 292 m/s (phase 2) and 250 m/s (phase 3) suggest that the three phases correspond to three separate infrasound ducts at different heights within the atmosphere^23^. The measured celerities match the expected values for tropospheric (phase 1), stratospheric (phase 2) and thermospheric (phase 3) waveguides. Thus we conclude that we have observed all three types of infrasound pathways on the seismo-acoustic recordings of the explosion. Below we will discuss the comparison of our measurements with acoustic ray tracing.

The dense spatial sampling of the infrasound field by the AlpArray seismic network reveals large zones in which no or only very weak seismo-acoustic arrivals were measured. This observation should, however, not be confused with classical shadow zones known for infrasound propagation^23^, as it’s still influenced by the seismo-acoustic coupling. Notably, the infrasound array IS26 of the CTBTO International Monitoring System, which is located near the border tripoint of Germany, Austria, and the Czech Republic, (near station GEC2 in Figs 1 and 3) falls into an area where no or only very weak seismo-acoustic signals were detected in this study. However, the acoustic signal was in fact detected by the very sensitive infrasound array IS26^24^. This demonstrates that acoustic energy may be present even though it does not map into the seismic recordings, because of the required seismo-acoustic coupling which may only be effective above a certain acoustic amplitude threshold^25^.

### Location and origin time

We obtained the origin time of the explosion by three independent approaches: Seismic grid search, acoustic grid search and modeled travel times, all based on the manual onset picks. The results from seismic and acoustic grid search agree within 2 seconds difference and determine the origin time on average to 03:11:45 UTC ±1 s. Due to the uncertainty of the underlying velocity estimates we do not speculate if the different origin times may have a physical explanation. The determination of the origin time depends on the seismic and acoustic velocities used during the grid search. Any false assumption may map into both the location and origin time. We tested seismic velocities between 5–6 km/s and acoustic velocities between 330–350 m/s and compared the resulting output of the grid search. The best fitting location is only weakly affected by choosing a different velocity, and varies around 1 km from ground truth. This indicates that the local velocity structure (in both ground and atmosphere) is rather homogeneous and that assuming a constant value is reasonable. Consequently, changing the propagation velocities only affects the origin time, with slower propagation resulting in earlier origin time. However, ground truth video records are available that rule out any origin time earlier than 03:11:44, UTC (K. Koch, personal communication).

Using the modeling results, that is subtracting modeled acoustic travel times from the first onset of the acoustic signal, results in origin times considerably earlier than estimated by the grid search approach (see Table 2). However, since bounce points are only modeled West and South of the explosion site, the results strongly rely on correct measures of the acoustic velocities and trajectories. As video material contradicts any origin time earlier than 03:11:44, we conclude that the ray tracing likely overestimated the true travel times, and that origin times based on ray tracing may not be reliable in this case.

### Signal polarities and amplitudes

For the nine seismic stations closest to the explosion site the first motion polarity of the the P wave shows a clear upwards motion (see Fig. S4), regardless of the azimuth. This is consistent with an isotropic source model as expected for explosive sources. The first motion polarity of the seismo-acoustic signals is less conclusive, yet it indicates a downward motion on several stations (see Fig. S5). This polarity would be in agreement with acoustic pressure pulses arriving from above ground, pushing down the earth surface and thereby locally inducing downward first motion. Still, local seismo-acoustic coupling conditions in the vicinity of a station might potentially obscure clear first motion polarities, which could be why we observe a less conclusive pattern for acoustic waves compared to the seismic waves.

In principle, the high spatial density of our measurements could allow to determine the attenuation of the infrasound waves on regional scale, especially in potential tropospheric ducts. However, seismic sensors measure ground velocity and the exact relation of ground velocity to air pressure changes is unknown and might be highly site-specific. We observe large scatter in the maximum amplitudes of the seismo-acoustic signal (Fig. 5). For any given distance range the measured seismo-acoustic amplitudes generally scatter within 1–2 orders of magnitude. Differences in seismo-acoustic amplitudes may be due to different attenuation properties along the acoustic raypath, in particular caused by potentially anisotropic attenuation due to winds^26^. Differences in seismo-acoustic amplitudes may also be due to local coupling conditions. The ray tracing results due not indicate strong differences in amplitudes, and the fluctuations we observe are likely due to specific seismo-acoustic coupling conditions at the individual seismic stations.

Generally, air-to-ground coupling is poorly understood, especially when it comes to relating seismic amplitudes to acoustic amplitudes^25–27^. Potential mechanisms for air-to-ground coupling are direct vertical forcing of the ground due to air pressure pulses^28,29^ and so called air-coupled Rayleigh waves^30^. The latter are generated if Rayleigh waves velocities in near surface layers are equal to the speed of sound or to the sweep velocity of the acoustic wave field^30^. The arrival times of the seismo-acoustic signals in this study are consistent with purely acoustic propagation and do not indicate any air-coupled Rayleigh wave as origin. Thus, we focus on the direct surface forcing by a pressure pulse. We suggest that this mechanism is responsible both for the primary creation of seismic waves at the explosion site as well as the creation of seismo-acoustic signals at each individual seismic station. However, very few studies exist that attempt to investigate this coupling mechanism in detail, in particular in the context of near-surface explosions. Generally, soft surface conditions will allow for more efficient seismo-acoustic coupling than stiff surface conditions. Several studies are available that investigate the efficiency of seismo-acoustic coupling after bolide/fireball explosions at high altitudes^27,31^. Such studies highlight that the coupling efficiency is affected by frequency^31^ and even more so by local site conditions^27,32^. To our knowledge no study investigates the seismo-acoustic coupling efficiency at the frequencies observed in this study (1–2 Hz). Additionally, there is still considerable discrepancy among different studies about the efficiency of air-to-ground coupling, ranging from efficiencies as low as 10^−5^ ^30,31^ to values of 0.5^27^, again likely due to site effects. Still, Edwards *et al*. provide a detailed analysis of air-to-ground coupling, and point out that in principle a scaling relation between seismic ground motion (in m/s) and air pressure changes (in Pa) can be established^27^, if the properties of the shallow subsurface are well known. However, such information (e.g. Vs_3_0) is unfortunately not available for the stations we used in this study. Other studies suggest that energy transfer from air to ground requires a soft and unconsolidated surface layer of at least 10 m thickness^32^.

In this study, seismo-acoustic amplitudes measured on temporary seismic stations are notably higher than amplitudes measured on permanent seismic stations at the same distance (see Fig. 5). Within 25–50 km from the source, the seismo-acoustic amplitude is on average more than 70 times higher on temporary AlpArray stations than on permanent stations. At larger distances, temporary stations still on average record 10 times larger seismo-acoustic amplitudes than permanent stations. Permanent seismic stations are commonly installed on hard rock in underground vaults and are carefully shielded from external disturbances, such as pressure variations. In particular the seismic stations of the Gräfenberg array just 10 km north of the explosion site are installed into solid limestone formations^16^. All permanent stations within 50 km from the explosion are part of the Gräfenberg array. Hence, we expect weak seismo-acoustic coupling, as evidenced in Fig. 5. In contrast, most of the temporary AlpArray stations are installed in near-surface structures such as buildings and within the top-most potentially soft surface layer. In particular, all temporary stations south of the explosion site are inside the soft Molasse unit. Consequently, these stations are potentially more sensitive to atmospheric perturbations, as they are both more exposed and situated in soft surface structures. Additionally, the AlpArray stations are generally less carefully shielded against pressure variations^33–36^. Both effects will allow for more efficient seismo-acoustic coupling into temporary stations than into permanent stations. This may explain the large variation of seismo-acoustic amplitudes in Fig. 5.

Thus, we only point out that despite the amplitude variations, between 0–200 km from the source (that is within the range of phase 1 = tropospheric ducting) seismo-acoustic amplitudes on average decay by approximately one order of magnitude per 100 kilometers (Fig. 5). This holds even if some of highest or lowest amplitudes are solely due to site effects. For distances larger than 200 km from the source, we cannot observe any clear trend. Generally, we do not account for potential azimuthal anisotropy. The large scatter in seismo-acoustic amplitudes renders any detailed analysis of the acoustic attenuation unreliable, yet we attempt to relate our observations to the findings of other studies. Le Pichon *et al*. derived an attenuation formula for infrasound from a synthetic dataset, assuming the presence of a stratospheric duct but no tropospheric duct^37^. At 180 km distance from the source they predict that without a tropospheric duct amplitudes are attenuated by 4–5 orders of magnitude due to geometrical spreading and dissipation of direct waves within the troposphere. Our seismo-acoustic observations indicate that within the troposphere attenuation may be significantly less if an efficient infrasound duct is present. However, the unknown and highly site-dependent seismo-acoustic coupling prohibits an in-depth analysis of the acoustic attenuation in this study.

To conclude we highlight that several studies suggest using the dependence of air-to-ground coupling efficiency on local material properties to turn seismo-acoustic observations into a tool to locally infer such properties of the near surface layers^32,38^. This requires simultaneous acquisition of pressure and seismic data, though. In turn, if site properties such as Vs_3_0 were known for all sites in this study, it could be possible to reconstruct the acoustic wave field from seismic records alone^27^.

### Infrasound propagation

Above we argued that phases 1, 2 and 3 likely correspond to infrasound ducting within the troposphere, stratosphere and thermosphere, respectively. We performed acoustic ray tracing to test our hypothesis and to explain the distinct spatial detection patterns that we observe for the three different phases. Phase 1 is predominantly observed at azimuths between 135–315° at distances up to 200 km (see Figs 3 and S3a). These detections coincide well with expected surface bounce points and expected celerities predicted by the numerical modeling (see Fig. 6), confirming that we observe infrasound waves guided within the troposphere. The corresponding turning height for the tropospheric reflections is less than 1 km. However, ray path modeling for several stations West/South of the explosion site apparently overestimates the acoustic travel times, resulting in unrealistically early origin times for the explosion (see Table 2). This indicates, that the model may underestimate celerities within the tropospheric duct or produce wrong trajectories. It should be noted that very shallow turning heights as observed here should generally interpreted with caution, being on the limits of what ray tracing can resolve. Towards south-west ray tracing also predicts tropospheric waves only up to 80 km from the source, while we still observe them at 160 km distance. Additionally, the modeling does not reproduce the narrow corridor of high-celerity detections north of the explosion site. Weather balloon data is available from a launch site 80 km north of the explosion site, however only for 2018-09-01 00:00 UTC (3 hours before the explosion). Using wind and temperature data measured by the balloon for the ray tracing we are able to reproduce some tropospheric reflections towards north, with very shallow turning heights of <1 km (see Fig. S6). Despite the three hours offset in time, this indicates that sharp but small-scale variations in the atmosphere that are not captured by the smooth ECMWF model might be responsible for the tropospheric detections north of the explosion site.

**Figure 7:**
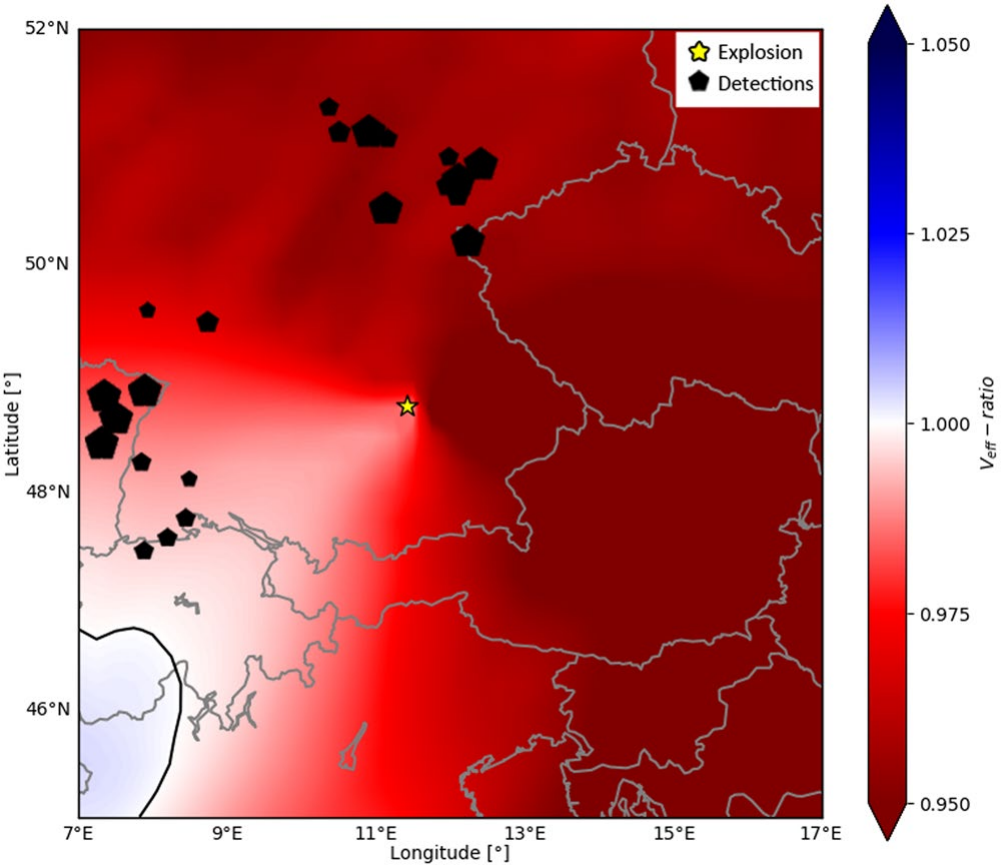
Map of the V_eff_-ratio. The color shows the expected V_eff_-ratio for propagation with respect to the explosion site, as if the respective point on the map was the hypothetical turning point of a stratospheric ray (effective sound speed at ground is the value at the explosion site into the respective direction). Note that in order to have a detection at the surface the corresponding turning point would be approximately halfway in between the detection point and the explosion site. The map clearly indicates that for the study area the ECMWF model only predicts V_eff_-ratios smaller than one. A star denotes the explosion site; positive detections are marked by black polygons.

We observe phase 2 in the azimuth range 240–300° (west) and 330–45° (north) (see Figs 3 and S3b) and at distances of 150–325 km from the explosion. Note however that within this distance range there is an azimuthal gap with little to no stations between 300–330° (north–west). Thus, we cannot state for sure if the two patches of stratospheric detections are separated or potentially connected all the way from west to north. In any case, we observe clear evidence for stratosphere-guided waves that are not at all reproduced by ray tracing (Fig. 6). The potential presence of a stratospheric duct is usually evaluated by the V_eff_-ratio, which is defined as the ratio between the effective sound speed at stratospheric altitude and the sound speed at ground level^37^. Stratospheric reflections are expected when the effective sound speed at height is faster than at ground, that is for V_eff_-ratio > 1. Figure 7 shows a map of the effective sound speed ratio V_eff_-ratio 15 minutes before the explosion (03:00 UTC), based on the ECMWF atmospheric model. Not knowing the exact stratospheric boundary, we chose the maximum effective sound speed within 35–60 km altitude as a measure for the stratosphere.

Figure 7 displays the V_eff_-ratio for our study area as if each respective point on the map was the hypothetical turning point of a stratospheric ray propagating radially outwards from the explosion site. Note that in order to have a detection at the surface the corresponding turning point would be approximately halfway in between the detection point and the explosion site. The map clearly indicates that for the study area the ECMWF model only predicts V_eff_-ratios smaller than one (except at the edge of the study area towards south-west. However, the corresponding potential surface bounce points would lie beyond our study area). This is because the effective sound speed at ground at the explosion site is always slightly larger than the effective sound speed at stratospheric heights in any direction and at any distance. Consequently, ray tracing should not predict any stratospheric reflections. Note, that this considerations about the spatial distribution of the V_eff_-ratio are based on a simplified 1D propagation of the rays in each direction. The actual modeling was done with fully three-dimensional propagation, which may slightly deviate from the simplified 1D path.

Our stratospheric detections north of the explosion follow a similar corridor as the tropospheric detections. As the atmospheric conditions are generally close to allowing stratospheric reflections with V_eff_-ratio close to 1, we speculate that small-scale atmospheric perturbations are responsible for both the tropospheric and stratospheric reflections towards north. Such perturbations might be particularly effective if located close to the infrasound source^39,40^. Similarly, the stratospheric detections towards west could be due to small-scale perturbations that are not included in the atmospheric model or due to gravity waves.

Phase 3 is predominantly detected in an azimuth range from 350–115° (Figs 3 and S3c), but it shows up only weakly on Fig. 2 and should be interpreted with caution. Based on the measured celerity and distances we suggest that phase 3 corresponds to thermospheric arrivals. The acoustic modeling, however, predicts thermospheric arrivals at all azimuths and at slightly different distances than observed (Fig. 6). For northwards and eastwards directions we detect phase 3 about 50 km closer to the source than expected. Few detections towards north-east even fall 100 km closer than predicted by modeling. Note, that the modeled celerity of all thermosperic arrivals increases with distance. This feature is for example reproduced by a group of measurements towards the East (in Austria) that show a similar increase with distance for the measured celerity and generally a good match between modeled and measured celerity, despite the 50 km offset in bounce points. This supports the interpretation of phase 3 as thermospheric. Generally, however comparing measurements to model results suggests that the thermospheric conditions on that day were not properly reproduced by the atmospheric model. Given that the effective sound velocity in the thermosphere always exceeds the effective sound velocity at ground level, thermospheric phases are always predicted by modeling. However, they are only rarely observed even on microbarographs due to the relatively low density of the atmosphere at high altitudes that strongly attenuates especially higher frequencies^41^. Gibbons *et al*. observed weak seismo-acoustic thermospheric arrivals on seismic arrays in Scandinavia^12^. Beside low celerity values they confirm that thermospheric arrivals consist of lower frequencies^26^. Assink *et al*. report similar effects after the 2017 North Korea nuclear test^13^. We cannot clearly identify such effects in our measurements, but we note that the potential thermospheric arrivals are no longer present for frequencies higher than 3 Hz, while tropo- and stratospheric detections are still well-visible (Fig. S1, supplemental).

Compared to a similar study by^6^ our observations are less in agreement with predictions by acoustic ray tracing, even though we calculated the infrasound propagation in the fully 3D atmospheric model. The discrepancy between observation and model calls for explanation. Small scale disturbances at low altitudes may not be captured by the smooth ECMWF models, but only in local measurements from e.g. weather balloons. Such measurements, however, are only taken every 12 hours and may not properly represent the state of the atmosphere for the time of interest. Regarding the discrepancy among modeled and observed stratospheric reflections we point out that the explosion occurred close in time to autumnal equinox. Studies highlight that at this time of the year there is little stratospheric wind, enhancing turbulence and small-scale wave activity in the middle atmosphere (stratosphere-mesosphere)^13^. In fact, the ECMWF model we applied does generally show weak stratospheric winds. This may allow for disturbances that are not accounted for in the atmospheric models, but only in local measurements e.g. from Lidar^42^. Such measurements, however, are only taken every 12 hours and may not properly represent the state of the atmosphere for the time of interest. During calm stratospheric conditions small-scale structures and wind shear layers can occur^39^, causing unexpected reflections and infrasound arrivals, such as our tropospheric and stratospheric detections north of the explosion site. Assink *et al*. point out that high frequency signals (>1 Hz) such as the ones we observe are more likely to be affected by small-scale structures^13^.

In turn this demonstrates the usefulness of dense spatial sampling of the infrasound wave field. Theoretically, the discrepancy between observations and models could be utilized to improve the underlying atmospheric models. Dense seismo-acoustic measurements may then eventually allow to infer atmospheric properties on several hundreds of kilometers scale^43^.

## Conclusions and Outlook

We observed strong seismic and seismo-acoustic signals generated by an explosion inside an oil refinery near Ingolstadt, Germany. Seismic phases are detectable up to 400 km from the source (extension of our study area), and seismo-acoustic phases are detectable up to 350 km from the source. The dense seismic station coverage of 40 km allowed to study the propagation of seismic waves and infrasound waves with high spatial resolution. We detected tropospheric, stratospheric, and potentially thermospheric reflections.

Our work demonstrates the usefulness of dense seismic networks to study both seismic and infrasonic wave phenomena in great detail. We highlight that such networks may also be valuable for atmospheric studies, in particular as dedicated infrasound sensors are usually rare. We demonstrate that on regional scale there might be considerable discrepancy between the modeled and the true state of the atmosphere. Especially temporary seismic installations are very sensitive to impulsive atmospheric perturbations, as e.g. generated by explosions. However, highly site-specific seismo-acoustic coupling complicates the interpretation of seismo-acoustic amplitudes. Ultimately, with a more precise understanding of seismo-acoustic coupling, it might be possible to infer the state of the atmosphere from seismic records. Even more so, if all seismic stations from the temporary AlpArray network (or comparable future networks) were equipped with additional pressure sensors - as was done in parts for the USArray - explosive events like the one from this work would provide an unprecedented wealth of information, potentially enabling us to invert for atmospheric properties over hundreds of kilometers^14^.

### Data and resources

Seismic waveform data used in this study is available for download at the European Integrated Data Archive (EIDA) at http://www.orfeus-eu.org/data/eida/index.html (last accessed August 2019). This study is based in part on data from the AlpArray seismic network^44^ which at the time of publication was not publicly available. Please visit http://www.alparray.ethz.ch (last accessed August 2019) for more details on data access and for more information about the AlpArray seismic network.

The ISC earthquake catalog search engine is provided at: http://www.isc.ac.uk/iscbulletin/search/catalogue/ (last accessed August 2019). The Swiss seismic event bulletin is available at: http://seismo.ethz.ch/en/research-and-teaching/products-software/earthquake-catalogues/ (last accessed August 2019). The Bavarian seismic event bulletin is available at: http://erde.geophysik.uni-muenchen.de/ (last accessed August 2019). The Austrian event bulletin is available via E-Mail request (seismo@zamg.ac.at).

ECMWF forecast models are available to registered users through the ECMWF Meteorological Archival and Retrieval System (MARS) or a python API: https://www.ecmwf.int/en/forecasts/datasets/archive-datasets (last accessed August 2019). The HWM14 model is accessible via a python interface: https://github.com/rilma/pyHWM14 (last accessed August 2019). The MSISE-00 model is accessible via a python interface: https://github.com/scivision/msise00 (last accessed August 2019). The GeoAc ray tracing code is kindly provided on GitHub: https://github.com/LANL-Seismoacoustics/GeoAc (last accessed August 2019).

All waveform data processing was done using the ObsPy toolbox^45^ versions 1.1.0^46^ and 1.1.1^47^. Codes, maps and graphs were created using^44^ Python 2.7.12 and Matplotlib 1.5.1.

For this study we used seismic data from several permanent seismic networks and we appreciate the continuous operation of these seismic networks by the responsible institutions (see references): BE net^48^, BW net^49^, C4 net, CH net^50^, FR net^51^, GE net^52^, GR net^53^, GU net^54^, HU net^55^, IU net^56^, IV net^57^, MN net^58^, NI net^59^, OE net^60^, OX net^61^, RF net^62^, SI net, SK net^63^, 533 SL net^64^, ST net^65^, SX net^66^ and TH net^67^.

## Supplementary information

Electronic Supplement
