# Supplementary material for: Rich observations of local and regional infrasound phases made by the AlpArray seismic network after refinery explosion
Source: Sci Rep. 2019 Sep 10;9:13027. doi: 10.1038/s41598-019-49494-2 (PMC6736795; doi:10.1038/s41598-019-49494-2)
Supplement: Supplementary file 1 — Electronic Supplement [file 41598_2019_49494_MOESM1_ESM.pdf]

Supplemental material to

Rich observations of local and regional infrasound phases made by the AlpArray seismic network after refinery explosion

Florian Fuchs, Felix M. Schneider, Petr Kolinsky, Stefano Serafin and Götz Bokelmann

Figure S1 - Record section for different frequency bands
Figure S2 - Measured acoustic arrival times & celerities
Figure S3 - Map of seismo-acoustic amplitudes for each phase individually
Figure S4 - Detailed view of seismic signal and P onset
Figure S5 - Detailed view of seismo-acoustic signal and onset
Figure S6 - Weather balloon data and corresponding modeling results

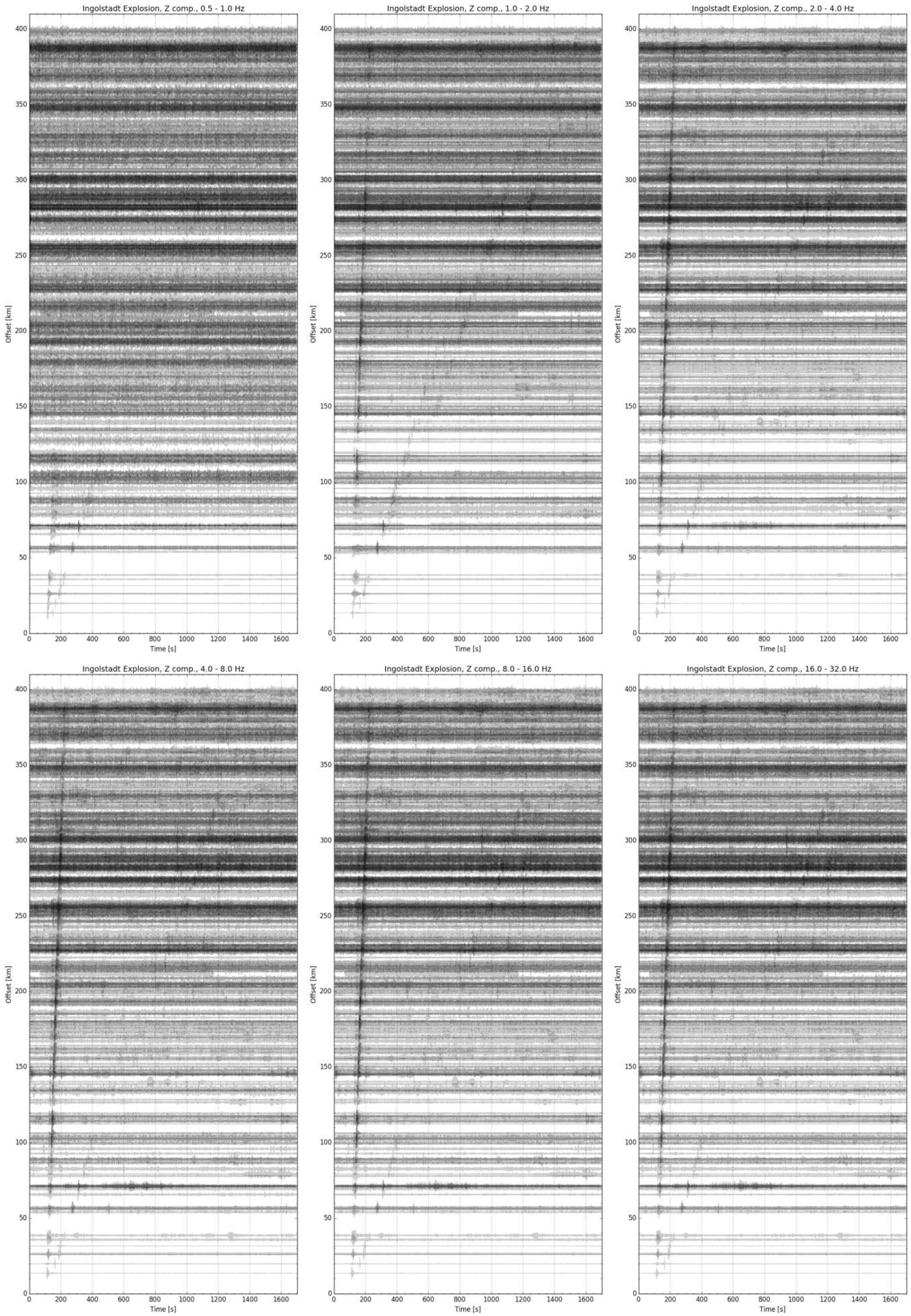

Figure S1 – Record section comparison for different frequency bands. Each panel shows all 400 vertical seismic traces without selection. The frequency range of the applied bandpass filter is indicated above each panel (increasing octave bands). All traces are scaled to their individual maximum.

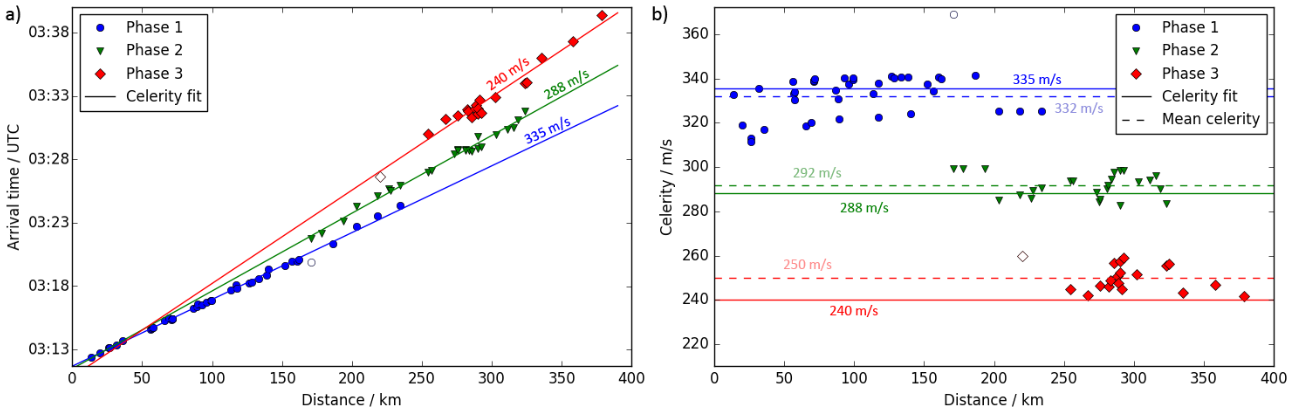

Figure S2 – Distribution of arrival times and respective celerities for the three acoustic phases. All values are read from the maximum vertical amplitude (PGV) within the respective time windows. Solid lines mark the fitted celerity for the respective phase. Dashed lines in panel b) mark the mean celerity as measured from arrival time and the estimated origin time 03:11:45 UTC. The measured celerities scatter within +/- 10 m/s around the respective mean. Outliers are not colored, and likely represents a false detection not associated with the explosion. Note that the distribution of measured celerities of phase 1 (blue) in panel b) may indicate two separate branches of distinct celerities: one with values of larger than 330 m/s and a second one with values from 315 m/s up to 320 m/s. Note also that the celerity fitted to the measured maxima of phase 3 (red) does not reproduce the same origin time as for phases 1 (blue) and 2 (green). Consequently, fitted and measured mean celerity do not match for phase 3 (red) in panel b).

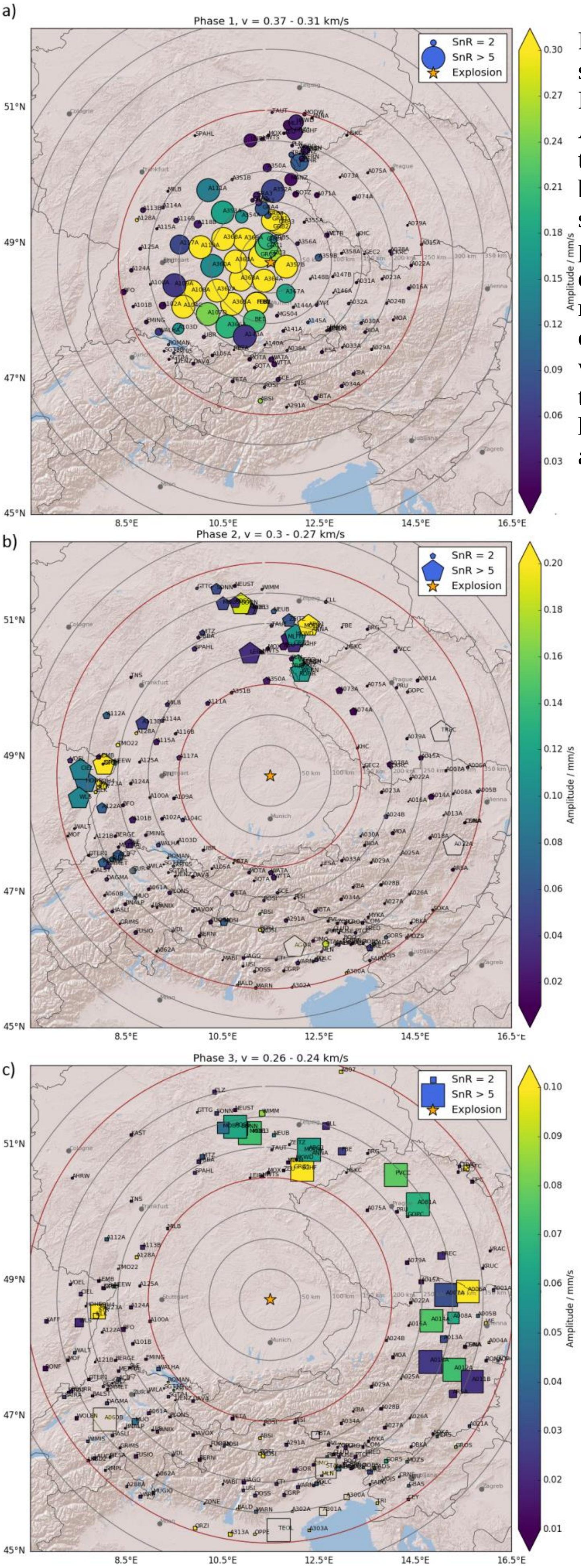

Figure S3 – Amplitude maps for each of the seismo-acoustic phases individually. Top: Phase 1, Center: Phase 2, Bottom: Phase 3. All Symbols are colored by amplitude (note the different color scales) and scaled in size by the signal-to-noise ratio (SnR). Note that some of the isolated high SnR markers for phases 2 and 3 might be false positive detections due to strong noise in the respective time window. We removed the color for these markers. The map image was created using the Matplotlib Basemap toolkit version 1.0.7: https://matplotlib.org/basemap/ (last accessed May 2019)

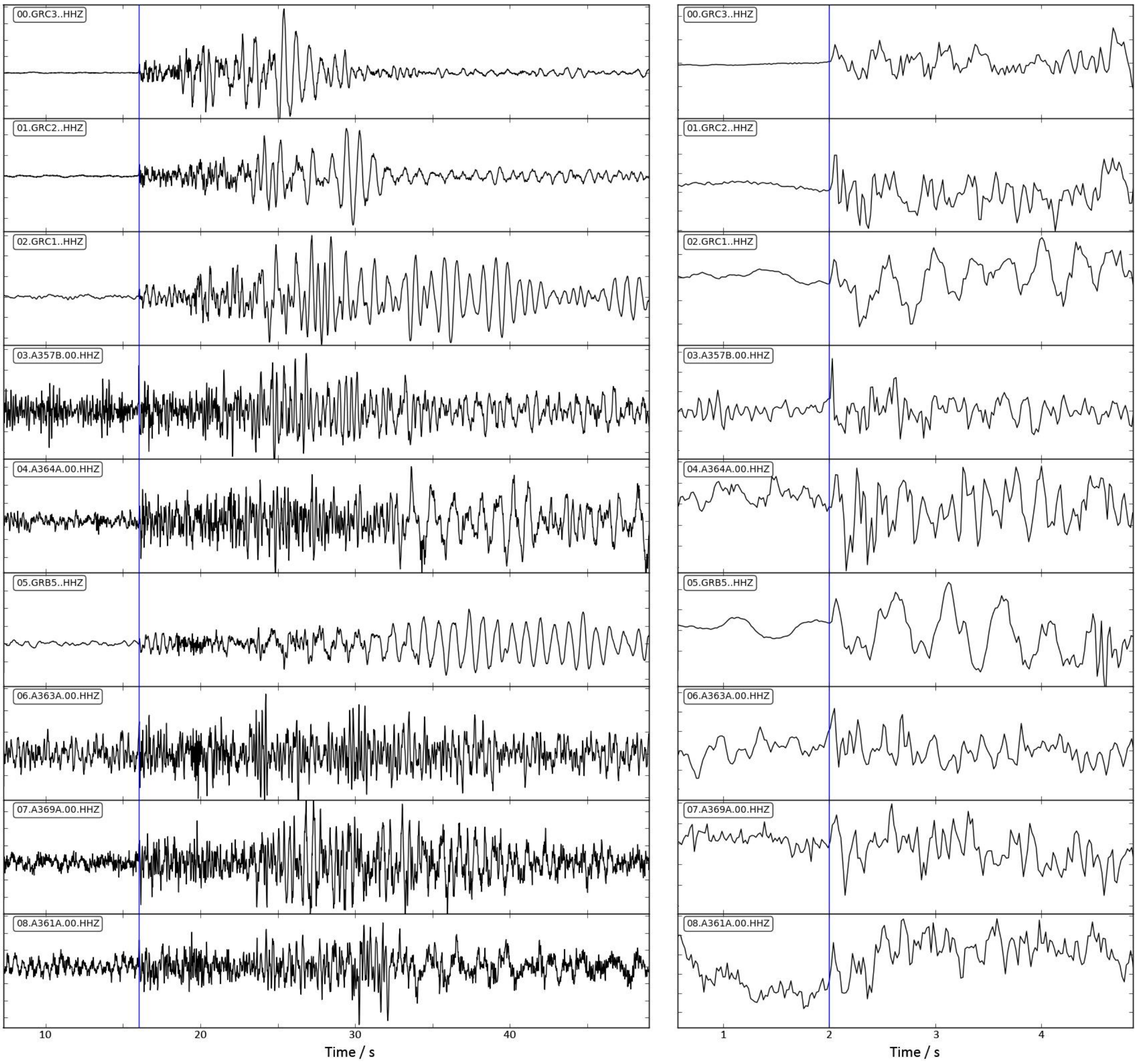

Figure S4 – Detailed view of the seismic explosion signal on the nine closest stations. Blue lines mark the onset picks. All traces are individually scaled in amplitude and shifted such that they align in time. Waveforms in the left panel were bandpass filtered between 0.5 -25 Hz, to suppress strong microseimic noise on several stations. The right panel shows a closeup of the first onset of the seismic P wave, bandpass filtered between 0.1 – 25 Hz.

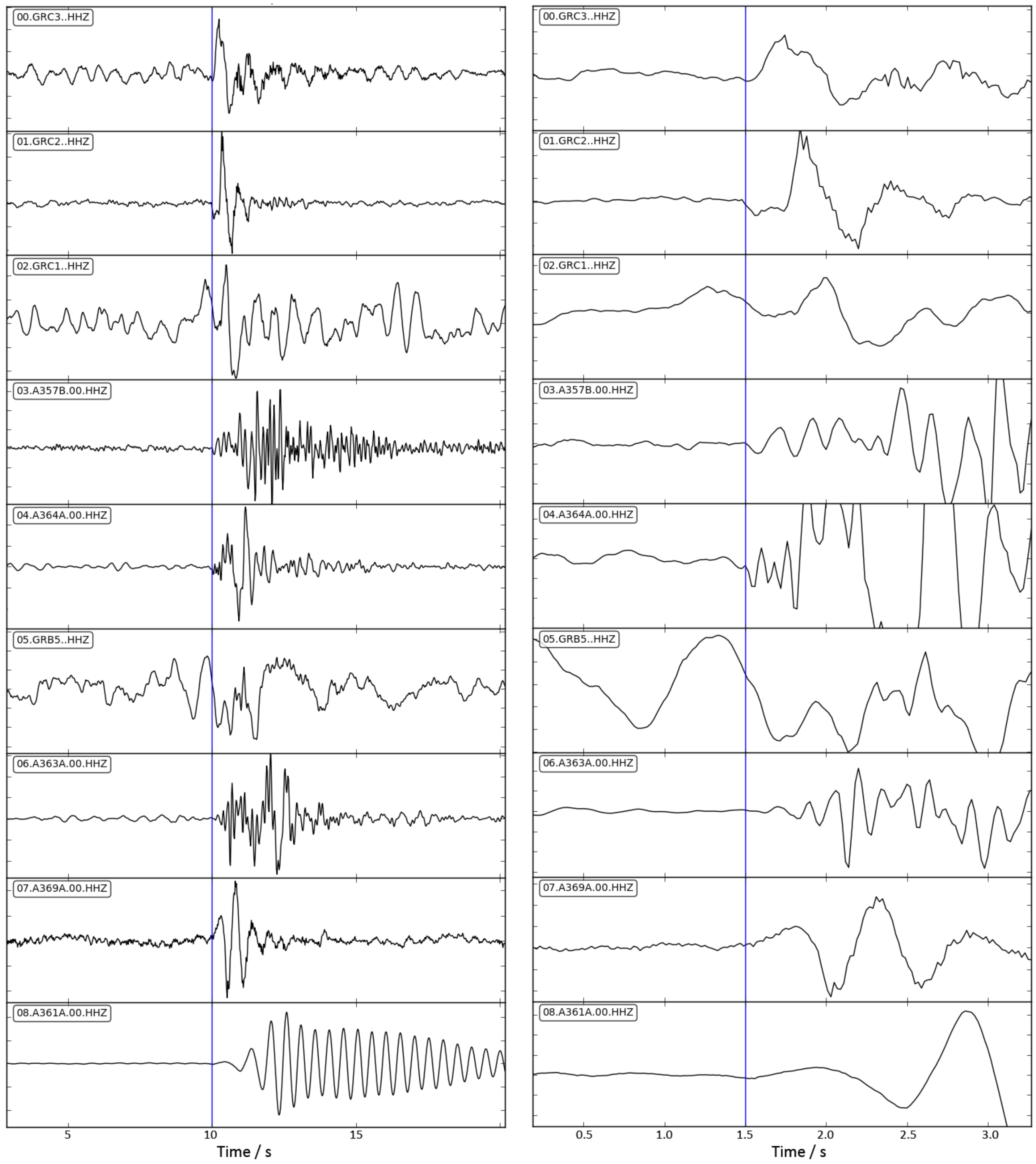

Figure S5 – Detailed view of the seismo-acoustic explosion signal on the nine closest stations. Blue lines mark the onset picks. All traces are individually scaled in amplitude and shifted such that they align in time. The left panel shows the entire signal, the right panel shows a closeup of the first onset of the seismo-acoustic wave. All waveforms are bandpass filtered between 0.1 – 25 Hz.

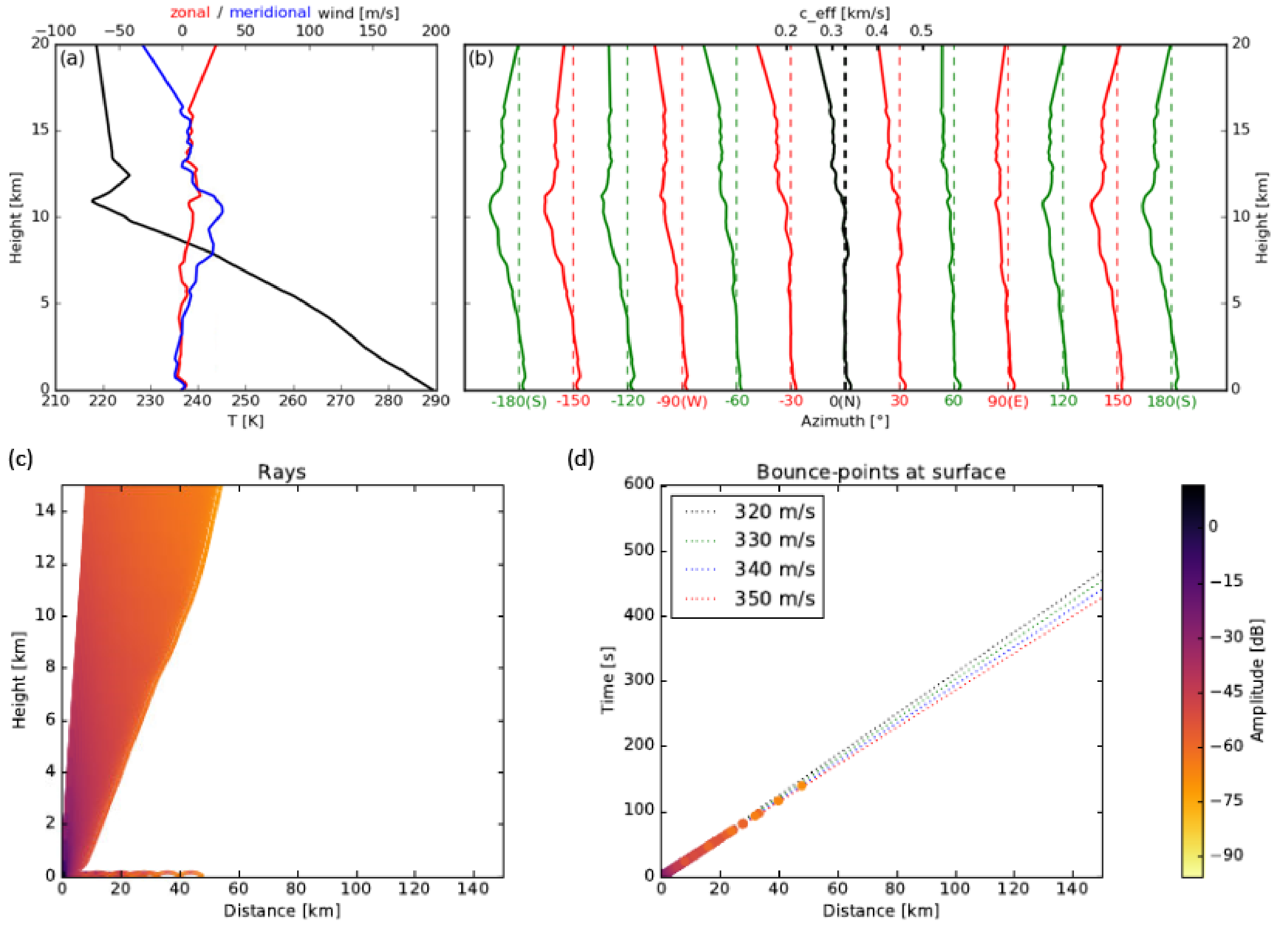

Figure S6 – Weather balloon data and corresponding modeling results. Panel (a) shows the atmospheric data acquired by a weather balloon at 2018-09-01 00:00 UTC above Kümmersbruck, 80 km north of the explosion site. Panel (b) displays the calculated effective sound speed for different azimuths. Panels (c) and (d) show the results of 1D raytracing in North direction based on this data, indicating a potential shallow duct that cannot be reproduced with ECMWF data, but potentially explains positive detections far north of the explosion site.
